# Supraclavicular Brachial Plexus Block for Challenging Anterior Shoulder Dislocations: A Case Series

**DOI:** 10.5811/cpcem.24850

**Published:** 2025-01-16

**Authors:** Michael Shalaby, Gregory Oliva, Christopher Raciti, Michael Rosselli, Oren Mechanic

**Affiliations:** * Herbert Wertheim College of Medicine, Department of Emergency Medicine and Critical Care, Miami Beach, Florida; † Mount Sinai Medical Center, Department of Emergency Medicine, Miami Beach, Florida

**Keywords:** regional anesthesia, supraclavicular brachial plexus, anterior shoulder dislocation, ultrasound

## Abstract

**Introduction:**

Emergency physicians frequently manage anterior shoulder dislocations (ASD). While there are many effective methods to reduce an ASD, adequate analgesia is imperative.

**Case Series:**

We used the supraclavicular brachial plexus (SBP) block to reduce ASD in three patients.

**Conclusion:**

The SBP block reliably anesthetizes the entire upper extremity, including the shoulder, by targeting all trunks and divisions of the brachial plexus. Complications are rare. Considering its ease of implementation and paucity of complications, the SBP block may be an effective means for reducing ASD.

## INTRODUCTION

Anterior shoulder dislocations (ASD) are a common orthopedic emergency, accounting for 45% of all joint dislocations and with a prevalence of 2% in the general population.^1^ First-time ASDs are typically traumatic in nature and most often occur in young male athletes or in domestic falls.^2^ Recurrent ASD occurs in up to 95% of patients, especially those who experience dislocation early or patients with associated Hill-Sachs deformity.^1^ Diagnosis is classically made via radiography. Adequate analgesia is imperative to the successful reduction of ASD. Options for emergency physicians include oral or intravenous analgesics, procedural sedation and anesthesia (PSA), intra-articular injection of anesthetic, and regional anesthesia (RA).

The supraclavicular brachial plexus (SBP) block is one of the oldest techniques for achieving upper extremity anesthesia. The brachial plexus is formed by the fifth cervical to first thoracic (C5-T1) nerve roots, which merge into three trunks (lower, middle, and upper) and descend toward the first rib. The SBP courses through the supraclavicular fossa encased within a sheath and containing all the nerve roots of the brachial plexus.^3^ To perform the SBP block, a linear ultrasound (US) probe is placed in the supraclavicular fossa, roughly in the medial to middle third of the clavicle, where the SBP is visible lateral to the subclavian artery and superior to the first rib and pleura (Image).

A needle (usually spinal) is advanced from lateral to medial, and local anesthetic is instilled within the SBP sheath or around it (Video). Injection near the “corner pocket,” a segment within the SBP sheath closest to the subclavian artery, helps to ensure dense anesthesia by ensuring the inferior trunk of the SBP is anesthetized.^3^ The SBP block anesthetizes the entire upper limb, including the shoulder, by targeting all nerve roots of the brachial plexus. In this case series, we demonstrate the utility of the SBP block for a challenging ASD reduction.

## CASE SERIES

### Case 1

An 18-year-old man presented to the emergency department (ED) with pain and a visible deformity of his right shoulder after falling off his skateboard onto an outstretched hand. Radiograph confirmed an ASD. The treating emergency physician administered 30 milligrams (mg) of intravenous (IV) ketorolac and 1 mg of IV hydromorphone, but the reduction was unsuccessful secondary to the patient’s pain. The patient’s care was subsequently handed off to the oncoming emergency team, who offered the PSA or SBP block. The patient chose a SBP block, which was performed with 15 milliliters (mL) 2% lidocaine, and his right shoulder was painlessly reduced on the first attempt. On follow-up the next day, the patient had a full return of strength and sensation and stated that the anesthesia lasted about six hours.

### Case 2

A 26-year-old man presented to the ED with pain and a deformity of his left shoulder after a motorcycle collision. A radiograph revealed an ASD. The treating emergency team initially attempted reduction with 30 mg IV ketorolac and 1 mg IV hydromorphone, but the reduction was unsuccessful secondary to the patient’s pain. The patient then consented to PSA, which was performed with 0.5 mg per kilogram each of ketamine and propofol, but this was also ineffective secondary to spasms of the patient’s shoulder girdle muscles. His care was transitioned to the oncoming emergency physician, who consented the patient to a SBP block, which was performed with 10 mL 2% lidocaine. The patient experienced complete anesthesia, and his left shoulder was easily reduced on the first attempt. On follow-up the next day, the patient had regained full strength and sensation in the left upper extremity and stated that the anesthesia lasted about three hours after discharge.

### Case 3

A 32-year-old woman presented to the ED after having awoken that morning with pain and a visible deformity of the left shoulder. The patient had had two previous ASDs, and a radiograph confirmed the diagnosis. The patient was offered a choice between PSA and a SBP block, but she preferred RA. A SBP was performed with 10 mL 2% lidocaine, and the patient’s left shoulder was reduced painlessly on the first attempt. On follow-up the next day, the patient regained full strength and sensation in the left upper extremity and stated that the anesthesia had lasted around four hours.

**CPC-EM Capsule**

What do we already know about this clinical entity?

*Anterior shoulder dislocation is common. Reduction can be performed with or without analgesia, with procedural sedation, or with a regional block*.

What makes this presentation of disease reportable?

*This is the first case series to report using the supraclavicular brachial plexus block to successfully reduce anterior shoulder dislocation*.

What is the major learning point?

*The supraclavicular brachial plexus block is relatively easy to perform, safe, and useful for managing anterior shoulder dislocation*.

How might this improve emergency medicine practice?

*Physicians now have another nerve block to help reduce anterior shoulder dislocation*.

## DISCUSSION

Multiple reduction techniques can be employed to reduce an ASD, primarily based on patient comfort and physician preference,^2^ but reduction without adequate analgesia portends a high failure rate.^4^ Attaining adequate analgesia for patients with ASD in the ED can be challenging, and patients who cannot be reduced will ultimately require hospital admission and open reduction. Options for analgesia include parenteral medications, PSA, intra-articular anesthetic, and RA. Parenteral analgesics, most often opioids in the case of joint dislocations, usually only dull pain and do not eliminate the noxious sensation from dislocation. Furthermore, opioids are associated with acute complications during reduction, such as respiratory depression, hypoxia, and nausea,^5^ and ultimately impart a higher failure rate than PSA.^4^

Procedural sedation and anesthesia, on the other hand, is effective at sedating patients and providing analgesia during reduction. However, PSA is time- and labor-intensive, requires cardiac and airway monitoring during the procedure and recovery, and mandates the presence of multiple personnel at the bedside. This, in turn, delays care for other patients in the ED. Moreover, PSA may mandate high doses of sedatives, prolong recovery times, and induce respiratory depression, nausea, vomiting, and hypotension.^5^ Notably, PSA does not paralyze but only relaxes muscles that actively resist reduction, which may still hinder reduction even in sedated patients. Intra-articular anesthetic can provide significant analgesia and is easy to perform via a landmark-based technique owing to the widened glenohumeral joint space in an ASD. Furthermore, patients managed with intra-articular anesthetic achieve reduction as often as patients who undergo PSA while experiencing fewer complications.^6^ However, intra-articular anesthetic does not anesthetize or paralyze spastic shoulder girdle muscles, and if it is not performed under sterile technique may result in septic arthritis.

The glenohumeral joint and intrinsic shoulder muscles derive their sensory innervation from the axillary, lateral pectoral, suprascapular, and lower subscapular nerves.^7^ By targeting these sensory nerves, the SBP block anesthetizes the glenohumeral joint capsule and alleviates pain induced by ASD. Additionally, disruption of the other cords of the brachial plexus paralyzes muscles of the upper extremity that actively resist reduction,^5^ making the SBP block an effective technique for reducing ASD. There are many favorable qualities of the SBP block that make it useful for the management of ASD as well as for other upper extremity injuries.

To begin, setup for the block is simple, usually requiring only a short linear probe (available on all cart-based systems) owing to the shallow course of the SBP 1–2 cm beneath the skin. Furthermore, patients can remain in a comfortable position with the arm adducted. Although the SBP block is associated with its own risks, adverse events are rare. Pneumothorax, for example, is a known risk of the SBP block, but its incidence has significantly decreased with the use of US. In a pooled analysis of more than 2,500 patients who received an US-guided SBP block, there were no instances of pneumothorax.^8^–^11^ The first rib acts as a backstop to the needle’s trajectory, so even if the needle is inadvertently directed past the SBP, a pneumothorax might be averted. Puncture of the subclavian artery is also minimized via US use and by the fact that the artery is distal to the needle’s intended trajectory. The rate of permanent neuropraxia is less than 0.05%.^12^

Transient hemidiaphragmatic paralysis, due to blockade of the C5 nerve root, which is a component of the phrenic nerve, occurs in up to 70% of patients but is usually well-tolerated in those without chronic cardiac or pulmonary disease.^13^ This is perhaps due to the compensatory effects of the contralateral hemidiaphragm and the ipsilateral “minor” muscles of respiration (sternocleidomastoid, scalene, and intercostals). Transient Horner syndrome is also a risk of the SBP block. As in any form of RA, local anesthetic systemic toxicity (LAST) is a serious adverse event consisting of central nervous system excitation with or without cardiac instability, culminating in seizures and even possibly cardiac arrest. However, common to most of these adverse events is a decreasing incidence with increasing operator proficiency. Lastly, physicians who employ RA for ASD should be aware that most patients will experience a return of sensation to the upper extremity hours after discharge from the ED, preventing emergency physicians from assessing for axillary nerve damage. Therefore, all patients with ASD should have close follow-up with an orthopedic surgeon.

Other RA techniques have been described for the management of ASD. The suprascapular nerve block anesthetizes only the posterior glenohumeral joint and paralyzes only the supraspinatus and infraspinatus muscles.^14^ Therefore, the suprascapular nerve block may not provide adequate analgesia for an ASD (which distends the glenohumeral joint anteriorly and inferiorly) and will not paralyze all the spastic shoulder girdle muscles, which hinders reduction. Yu et al demonstrated that the retroclavicular approach to the infraclavicular region, an infraclavicular brachial plexus block, is a feasible option for ASD.^5^ However, the needle’s blind trajectory behind the clavicle followed by a narrow path between the pleura and the axillary artery makes less experienced users of RA hesitant to perform this block.

Lastly, the interscalene block, which targets the C5–C7 brachial plexus roots in the middle of the neck, is a more commonly used technique for ASD. However, in our opinion the interscalene block is more difficult to perform than a SBP block. First, the target nerves are smaller and more challenging to localize. Additionally, probe manipulation for an interscalene block requires that the physician maintain a steady grip on the probe farther up the neck while performing the block, as opposed to the SBP, which allows the physician to rest the probe in the supraclavicular fossa. Moreover, the interscalene block has been shown to carry a slightly higher risk of permanent neuropraxia.^15^

Lastly, all emergency physicians who perform regional anesthesia should be aware of the potential for LAST, a potentially fatal complication in which anesthetic is absorbed into systemic circulation.^16^ Local anesthetics cause toxicity by blocking sodium, calcium, and potassium channels in cardiovascular and neural tissue, culminating in the most extreme cases in coma, seizures, and cardiovascular collapse. Physicians can prevent LAST by placing all patients on a cardiorespiratory monitor prior to performing a nerve block. Early warning signs may include hypertension, dysrhythmias (brady- or tachycardia), subjective paresthesias, or perioral tingling. Patients who do experience LAST should be treated immediately with intralipid emulsion according to the American Society of Regional Anesthesia guidelines.^17^

## CONCLUSION

Considering its ease of implementation, low rate of complications, and the dense anesthesia and upper extremity paralysis that it imparts, the SBP block is practical for the reduction of ASD. In each of the patients in this case series, SBP blockade allowed for quick, painless, and uncomplicated reduction. More significantly, two of the three patients likely would have required surgery had reduction failed despite conservative management.

## Supplementary Information

Video Performance of a left-sided supraclavicular brachial plexus block. The left side of the screen is medial, the right side is lateral. With a high-frequency linear probe oriented obliquely in the supraclavicular fossa, the supraclavicular brachial plexus is immediately lateral to the subclavian artery.

## Figures and Tables

**Image:**
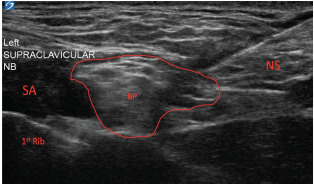
Ultrasound video still of supraclavicular brachial plexus block, with the brachial plexus sheath outlined in red. *SA*, subclavian artery; *BP*, supraclavicular brachial plexus; *NS*, needle shaft.
